# Real-time loudness normalisation with combined cochlear implant and hearing aid stimulation

**DOI:** 10.1371/journal.pone.0195412

**Published:** 2018-04-04

**Authors:** Dimitar Spirrov, Maaike Van Eeckhoutte, Lieselot Van Deun, Tom Francart

**Affiliations:** 1 ExpORL, Neurosciences, KU Leuven - University of Leuven, Leuven, Belgium; 2 Department of Otorhinolaryngology, Head and Neck Surgery, University Hospitals Leuven, Leuven, Belgium; Center for Healthy Start Initiative, NIGERIA

## Abstract

**Background:**

People who use a cochlear implant together with a contralateral hearing aid—so-called bimodal listeners—have poor localisation abilities and sounds are often not balanced in loudness across ears. In order to address the latter, a loudness balancing algorithm was created, which equalises the loudness growth functions for the two ears. The algorithm uses loudness models in order to continuously adjust the two signals to loudness targets. Previous tests demonstrated improved binaural balance, improved localisation, and better speech intelligibility in quiet for soft phonemes. In those studies, however, all stimuli were preprocessed so spontaneous head movements and individual head-related transfer functions were not taken into account. Furthermore, the hearing aid processing was linear.

**Study design:**

In the present study, we simplified the acoustical loudness model and implemented the algorithm in a real-time system. We tested bimodal listeners on speech perception and on sound localisation, both in normal loudness growth configuration and in a configuration with a modified loudness growth function. We also used linear and compressive hearing aids.

**Results:**

The comparison between the original acoustical loudness model and the new simplified model showed loudness differences below 3% for almost all tested speech-like stimuli and levels. We found no effect of balancing the loudness growth across ears for speech perception ability in quiet and in noise. We found some small improvements in localisation performance. Further investigation with a larger sample size is required.

## Introduction

There is currently a large number of people who use a cochlear implant (CI) together with a contralateral hearing aid (HA), as a result of relaxed CI candidacy criteria. The combination of acoustical stimulation by the HA and electrical stimulation by the CI is known as bimodal stimulation. Although there is individual variability, bimodal users generally have better speech intelligibility in quiet and in noise when they use both devices compared to the CI alone [1].

However, this improvement is sometimes lower than expected due to limiting factors associated with the used devices [2]. Even similar components like compressors are often different in the CI and the HA [3]. The differences between the modes of stimulation and the processing in the devices result in differences in loudness growth at both ears [4, 5]. Loudness growth relates the loudness to the sound intensity level. This means that even when sounds of the CI and the HA are balanced in loudness for a certain stimulus level, the loudness of the CI and the HA can be quite different at another level. This can lead to unbalanced sound perception [6] and can limit sound localisation performance [7].

To address the problem of different loudness growth functions, a loudness normalisation algorithm, called SCORE, was developed by Francart and McDermott [8]. For each segment of sound, this algorithm estimates the loudness caused by the CI and by the HA. The loudness of the CI is calculated based on the loudness model of McKay [9] and that of the HA is based on the model of Moore and Glasberg from 1997 [10]. For each device, based on the microphone signal, the algorithm also computes the loudness for a normal hearing subject. This loudness is used as a reference, which serves as the loudness target. Given that the loudness target is computed at each side, SCORE does not require communication between the devices. Based on the differences between the loudness target and the loudness caused by the CI or HA, the overall gain of the HA and the electrical current stimulated by the CI are adapted. As such, the algorithm returns the loudness growth to normal and automatically balances the HA and the CI for a frontal signal with any intensity and frequency content. Also, as such SCORE restores the interaural loudness difference, because for sounds coming from an angle different from 0 degrees, the targets at both sides will be different. The loudness target at the side where the sound comes from, will be higher than the loudness at the contralateral side.

The SCORE algorithm was implemented in Matrix Laboratory (MATLAB) computing environment and was evaluated in six bimodal listeners for speech intelligibility in quiet and in noise and in two listeners for localisation [11]. An improvement was found for sound localisation performance and for speech intelligibility in quiet for soft phonemes, but not for speech in noise.

Since the algorithm of Francart and McDermott was tested with an off-line implementation, the stimuli had to be preprocessed. Individual head related transfer functions were not considered. Also, spontaneous head movements that change loudness differences between devices and may be important for localisation were not taken into account. Therefore, a real-time version of SCORE was needed. Here, the computational complexity of the acoustic loudness model is a challenge. Therefore, it was necessary to simplify the acoustical models for real-time implementation.

On the real-time implementation of the normal hearing loudness model, there are some studies [12, 13]. These studies either used spectral peaks or non-uniform sampling of the spectrum of the signal. Then, both studies used the intensity differences between consequent time frames and computed the excitation pattern only after these differences were above a certain threshold. Therefore, the group delay caused by these algorithms depends on the incoming speech, making them sub-optimal for implementation in hearing devices.

An important parameter to consider is the loudness target. There is an ongoing discussion as to whether loudness growth should be restored to normal or not. It has been argued [14] that for listeners with substantial sensorineural hearing loss, for better audibility, we should provide comfortable loudness across all frequencies. Some fitting prescriptions aim for this [15]. Also, at the electrical side, there is evidence that lower intensities should be (almost) equally loud as higher intensities [16, 17]. SCORE allows investigating the effect of alternative loudness targets. For instance, we can enhance the loudness target compared to normal hearing loudness for lower intensities, in order to make the consonants almost equally loud as the vowels.

A number of studies [3, 7, 18, 19] investigated the effect of balanced loudness on speech intelligibility. However, the CI and HA were loudness balanced only for a number of intensities and/or frequencies. In contrast, SCORE enables us to study the effect of balancing the entire loudness growth functions on speech intelligibility and localisation. We assume that maintaining almost equal loudness for low and high intensities will disturb localisation since it reduces the interaural loudness difference (ILoD). Note that we use a slightly different abbreviation to distinguish from the interaural level difference (ILD), which is a physical phenomenon caused by the head-shadow effect. To improve localisation, we should enlarge ILoD by changing the loudness target in the opposite direction, which means reducing the loudness target for lower intensities.

This study had three objectives. First, to simplify the acoustical loudness models and to validate them. Second, to implement SCORE in a real-time system and to test it in bimodal users, both with a linear and with a compressive hearing aid. Third, to change the loudness targets at the two sides to assess the importance of loudness cues on speech intelligibility and localisation.

## Real-time implementation: Methods and validation

The major difficulty for a real-time algorithm is the implementation of the loudness models: a normal hearing loudness model that is used as a reference and a loudness model that computes the HA caused loudness and accounts for the degree of hearing impairment. The models use a number of equivalent rectangular bandwidth (ERB) filters that are level dependent [20] (a landmark article). It is challenging to compute a number of ERB filters on-line. In the models of Moore and Glasberg, loudness is a result of a multiplication of ERB filters and the intensities from the fast Fourier transform (FFT). It is also difficult to perform this multiplication on-line.

The loudness models were implemented and validated in Simulink (The Matworks, Natick, MA, USA). The execution time of the acoustical loudness models depends on two factors. First, the number of equivalent rectangular bandwidth (ERB) [20] filters and second, the number of FFT bins. In order to make the models faster we can reduce either of them. We know that speech signals have more power at lower frequencies [21]. Given that the cochlea has a logarithmic frequency organisation [22], whereas FFT provides a linear frequency spacing, it seems appropriate to group high frequency FFT bins. We tested three quasi-log FFT bin combinations in order to achieve bands that represent the distribution of the ERB filters more closely [23]. Such a combination of bins and consecutive summation of the bin power is often used for the design of HAs [24, 25]. The combinations resulted in 19, 13 or 8 frequency bands. We used a time window of 8 ms, a sampling rate of 16 kHz, and 128 FFT bins (64 bins until *π*/2), which means a bin spacing of 125 Hz. For the configuration with 19 bands, we started grouping bins from 1 kHz. Therefore bands 1 to 8 were identical to the first 8 FFT bins. For each band from 9 to 12, we grouped 2 bins. For bands 13 to 16, we grouped 4 bins, then, for 17 and 18, we grouped 8 bins. Finally, for band 19, we grouped 16 bins.

In the other two configurations, with 13 and 8 frequency bands, we started grouping bins for even lower frequencies, more specifically from 0.5 kHz and from 0.25 kHz. We also tested ERB step sizes of 1, 2 and 3 ERB numbers.

The rest of the loudness model implementation followed Moore 1997 [10]. Only for the middle-ear transfer function, we used that of Moore 2004 [26], similar to the off-line implementation of Francart and McDermott [8].

### Validation of the simplified models

For each of the three tested FFT configurations and ERB step sizes we compared the computed loudness to that of the original MATLAB implementation. We did this for three different hearing losses, one flat of 60 dBHL, one ski slope based on the data of Byrne [27], and one representative for the residual hearing of bimodal listeners using the data of Yoon [28]. Also, for the normal hearing loudness model, we compared the implementations when no hearing loss was present.

### Stimuli used for the validation

We used five stimuli, of which four were speech or speech-like: 1) a white noise of 1s filtered according to the long-term average speech spectrum (LTASS) from Byrne [21]; 2) a 2 s fragment of the international speech test signal (ISTS) from Holube [29]; 3) a 2 s fragment of a Swedish competing male talker from the story ‘The north wind and the sun’ (IPA, 1999); 4) one 3.9 s sentence from a Dutch female speaker from the LIST speech material [30]; 5) a 1 s frequency sweep (250–8000 Hz). For each stimulus we tested five presentation levels from 50 to 90 dBSPL in steps of 10 dB.

### Metrics for the validation

We compared the instantaneous loudness differences between the original MATLAB implementation and four Simulink implementations (the three FFT configurations above plus one with 64 bins that was used as a reference). For each signal frame of 8 ms we computed the loudness difference as a percentage of the loudness of the MATLAB model. Loudness differences were considered as outliers following two rules: first, if the MATLAB model loudness was below 0.1 sones and second, if the ratio between the loudness of the original and simplified model fell outside of the 99% confidence interval. The execution times were measured and the time reduction compared to the Simulink implementation with 64 bins was calculated.

### Results from the validation

**Fig 1:**
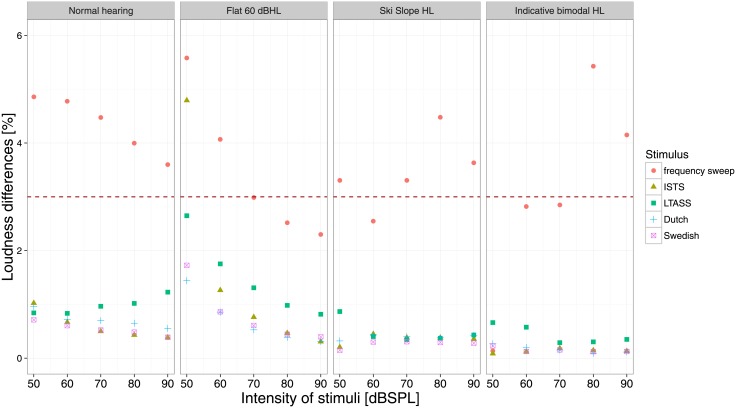
Median loudness differences. The dashed line shows the just noticeable loudness difference for noises, based on Allen (1997) [31].

The loudness differences were more influenced by the ERB step size than by the combination of bins. The overall trend was that the difference decreased exponentially with decreasing ERB step size. Also, as expected, the configuration with 19 bands yielded the smallest loudness differences. Therefore, the configuration with 19 bands and ERB step size of 1 was selected for further evaluation. The loudness differences for the selected configuration are shown in Fig 1.

Compared to the frequency sweep, the speech and speech-like stimuli showed smaller loudness differences. There was only one case (60 dB HL flat hearing loss and 50 dB SPL ISTS) where the median loudness difference was above the just noticeable loudness difference of 3% for noises based on the work of Allen [31]. However, all participants in the study (see below) had worse hearing than 60 dBHL. Therefore, for speech stimuli, we do not expect a substantial effect from the simplifications of the models. The time reduction for the three configurations is shown in Table 1.

**Table 1:** Execution time as a percentage of the baseline.

number of bands	normal hearing model	hearing impaired model
19	22.85%	22.21%
13	14.61%	14.22%
8	8.71%	9.39%

Execution time in % with respect to the time needed to execute the models without bin combination.

We were able to execute the complete algorithm with loudness models, based on 19 bins and 30 ERBs, with step size 1, in a real-time target xPC system. The system is used as a quick prototyping tool.

## Experiment 1: Normalising loudness growth

### Methods

In order to assess the effect of the model simplifications and the implementation of the real-time SCORE algorithm, we tested it in bimodal listeners.

#### Subjects

In total, nine native Dutch speakers (six male, three female) participated in either one of the experiments. All subjects were tested on speech perception in quiet. In the first experiment, six of them were tested on speech perception in noise; four were tested on localisation. One of the subjects (S2) had strong fluctuating tinnitus at the HA side. More information about the subjects is given in Table 2.

**Table 2:** Information about the subjects.

Subject	Age (years)	CI use (months)	CI side	Aetiology	Participants in experiment
S1	73	104	Left	genetic	1 and 2
S2	65	22	Left	noise induced	1
S3	68	33	Left	unknown	1 and 2
S4	85	9	Right	sudden	1 and 2
S5	65	41	Left	genetic	1
S6	24	23	Left	progressive	1 and 2
S7	17	27	Left	unknown	1 and 2
S8	34	14	Left	progressive	1
S9	75	15	Left	progressive	2

“Age” is given in years at the time of the first participation. “CI use” is implant use in months at the time of the first participation.

**Fig 2:**
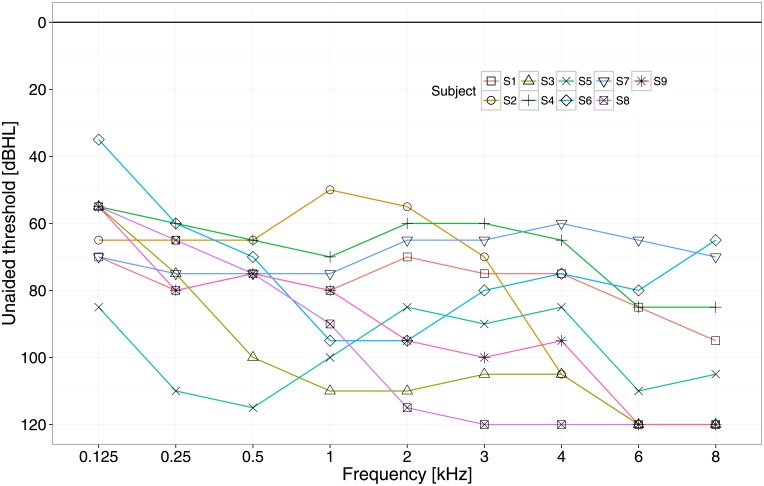
Unaided audiograms. Pure-tone unaided audiograms of the non-implanted ear at the time of the first participation.

The unaided audiograms of the hearing aid side are shown in Fig 2.

High frequency residual hearing was preferable to test a potential effect on localisation, given that bimodal listeners only have access to interaural level differences [11]. Therefore, we tested less people for localisation than for speech intelligibility.

The experiments were approved by the ethics committee of UZ Leuven. All subjects signed a declaration of informed consent before the experiments. Their travel costs were reimbursed.

#### Equipment

**Fig 3:**
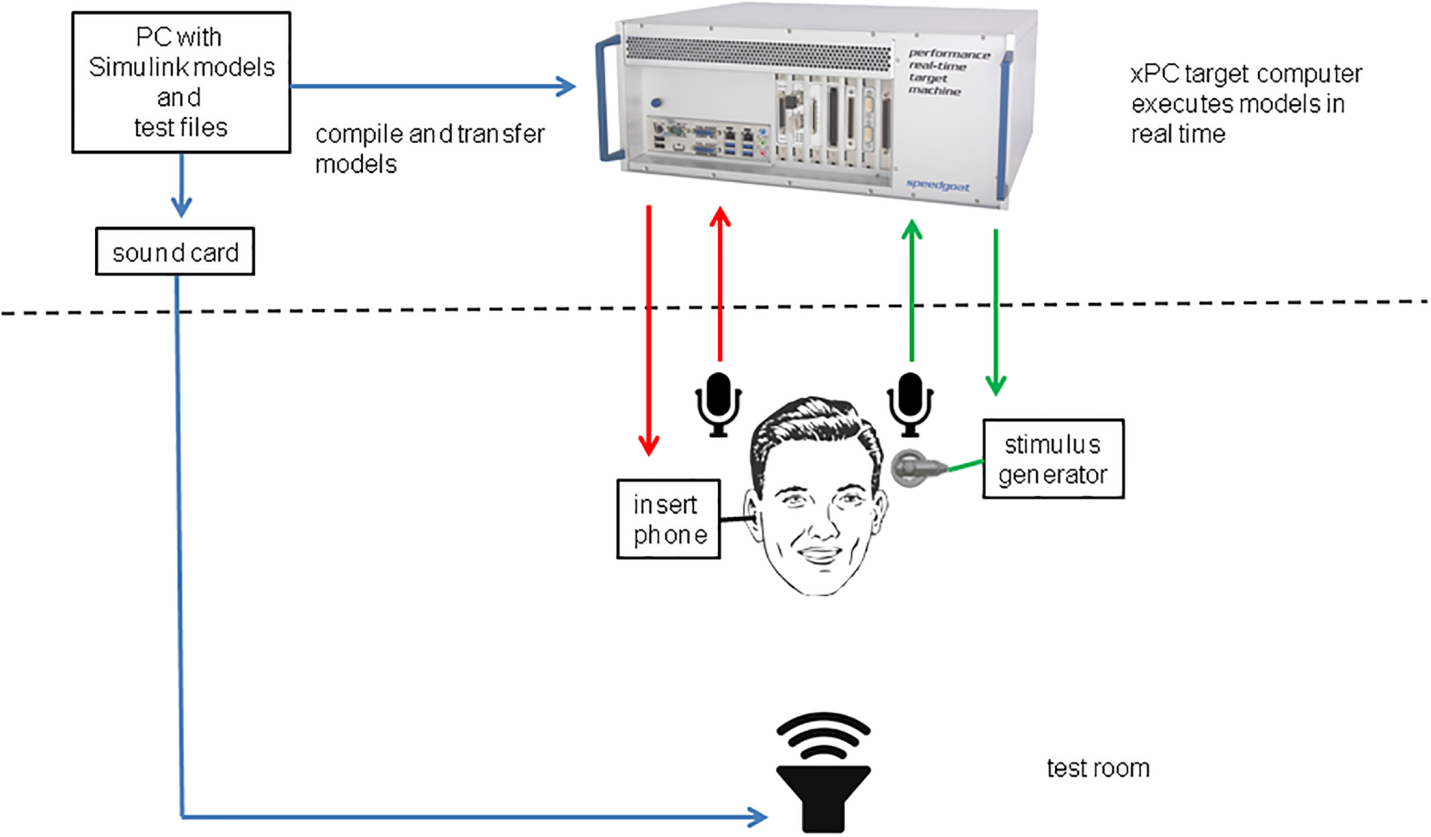
Experimental setup. A real-time system was used for HA and CI processing.

The experimental setup is shown in Fig 3.

We used a regular PC with APEX3 [32] and MATLAB/Simulink software (The Matworks, Natick, MA, USA). The computer was connected to three RME Multiface II sound cards (Audio AG, Haimhausen, Germany). One card was used for the speech perception experiments; the other two for the localisation test. The cards were driving one Genelec 8030A (Genelec Iisalmi, Finland) loudspeaker at 0° for speech intelligibility tests or an arc of 13 Fostex 6301B (Foster Electric Co., Ltd, Tokyo, Japan) loudspeakers from -90 to +90 degrees in steps of 15 degrees for the localisation test. The distance between the loudspeakers and the test subject was 1 meter.

For the CI stimulation we used a Simulink model of a cochlear implant (ACE strategy) provided by Cochlear Ltd, which means that the CI processing was identical to that of the clinical devices. All preprocessing options (AGC, ADRO, beam-former, etc.) were switched off. On the hearing aid side we used a linear HA model implemented as a 129-taps digital filter in Simulink. The filter coefficients were calculated from the HA insertion gains. The electrical and acoustical parts of the SCORE algorithm [11] were implemented as Simulink models. All described models were compiled and then executed on a real-time target xPC system (Speedgoat GmbH, Liebefeld, Switzerland) with Intel i3 dual core 3.3 GHz processor.

The stimuli at the HA side were presented using an ER-3A insert phone (Etymotic Research, Elk Grove Village, IL, USA). For the electrical stimulation, a Cochlear StimGen box provided by Cochlear Ltd. was used to connect the xPC target system to the participant’s implant. An oscilloscope was used to measure the time difference between the acoustical and the electrical stimulations (see below).

#### Calibration

Since tests were carried out in free field, we used a manikin CORTEX MK2 (Metravib, Limonest, France) for the calibration. The CI model was calibrated according to the recommendations of Cochlear Ltd. The slope of the loudness growth function of the electrical loudness model [11] was held constant. The electrical loudness model has a calibration parameter to give results in sones. This parameter was adapted until the additional current units from SCORE were zero or negligibly small. This means that SCORE was inactive for a comfortable loudness level. The HA model was calibrated by setting a broadband gain to match the output for a 60 dBA broadband noise presented in free field. The acoustical loudness model was calibrated by matching 60 dBA broadband noise to the recorded level in the xPC system.

We know that it takes about 1.5 ms for the acoustical signal to travel from the insert phone to the auditory nerve [33]. However, at the CI side the auditory nerve is directly stimulated. Therefore, we delayed the CI channel with 1.5 ms.

#### Stimuli

Speech perception in quiet was measured with consonant-vocal-consonant words of the Dutch NVA lists spoken by a male speaker [34]. Words were presented at 50 and 65 dBA. Each list has 12 words. The first word is a training word. We used one NVA list at each level.

Speech perception in noise was tested with the Dutch LIST sentences [30], spoken by a female speaker. Each list consists of ten sentences. The noise was a male competing talker in Dutch reading the story “The north wind and the sun” from IPA, 1999. Sentences were presented at a fixed level of 60 dBA.

For the localisation experiment we used a broadband telephone alerting signal of 250 ms [35]. The short stimulus was used in order to avoid the subjects turning their head while the stimulus was playing. The stimulus was presented at 65 dBA (±5 dB roving). Each level was presented once from each loudspeaker in a random order, which means 39 presentations (13 loudspeakers x 3 levels).

#### Procedures

First, to set the HA gains, we measured the pure tone audiometric thresholds. Second, we calculated the gains using the NAL-RP rule [36]. To avoid stimulation of possible dead regions, we set the gains to zero or highly reduced them where the thresholds were worse than 100 dBHL. In order to account for the real ear insertion gain, we measured the aided thresholds in free field. The insertions gains were adjusted until we reached the prescribed target with a maximum difference of 10 dB. This was not possible for one subject (S2) at two frequencies (125 and 250 Hz), where the difference was 17 dB. Third, we fine tuned the gains based on sound quality assessment. In general, this resulted in a decrease of high frequency gains, most probably due to dead regions in the cochlea. The CI was fitted according to the subject’s clinical MAP. Finally, without SCORE, we loudness balanced the HA and the CI, by adjusting the overall gain of the HA based on 60 dB SPL LTASS.

After the fitting subjects were made familiar with SCORE by listening to an audiobook for 10 minutes while SCORE was active.

Speech perception in quiet was tested in three listening conditions (CI only, HA only, and CI+HA) and two processing configurations (with and without SCORE). In order to maintain potential familiarisation with SCORE after the audiobook, all tests with SCORE were conducted first. For each block of tests (with and without SCORE), the listening conditions were presented in a random order.

Speech perception in noise was measured for the CI+HA listening condition only. Each test started at an initial signal-to-noise ratio of +5 dB. Based on key words recognition, the intensity of the masker was adapted with a 1up/1down procedure in steps of 2 dB. Final result was determined as the mean of the last 6 trials. To avoid possible procedure learning effects, we tested with SCORE until the results was decreasing. Then we did two measurements for both configurations (with and without SCORE) in a random order.

Sound localisation was tested in the CI+HA listening condition, with and without SCORE. Before testing, we did loudness balancing for each configuration, with the localisation stimulus (except for subject S1) by frontally presenting the localisation stimulus and changing the overall gain of the HA, if needed. During the localisation experiment the subjects had to identify the loudspeaker the sound came from. To avoid learning effects, listeners were trained with SCORE until the root mean square (RMS) error was decreasing. Then we did two test runs for each configuration, in a random order. In each run, the stimulus was presented three times from each loudspeaker, in a random order.

#### Statistical analysis

For speech intelligibility in quiet, we first averaged the test and retest results from the two retested subjects. Second, we transformed the results from percentage to rationalised arcsine units (RAU) [37]. On the RAU results we fitted an ANOVA model with three factors: 1) listening condition (CI only, HA only, CI+HA), 2) stimulation level (50 or 65 dBA), and 3) processing configuration (with or without SCORE).

For speech intelligibility in noise, we averaged test and retest results and then performed a Wilcoxon signed-rank test on the SRT results with versus without SCORE.

Since we tested a few subjects for localisation, we did not fit an ANOVA model on the results. However, we had many presentations for each subject. Therefore, we used a RMS error, within subject, to assess the results with or without SCORE.

### Results

#### Speech in quiet

**Fig 4:**
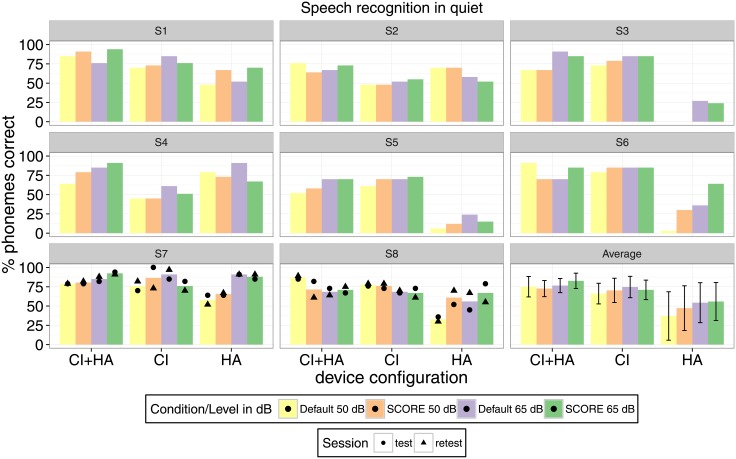
Speech intelligibility in quiet. Dots and triangles indicate test and retest results, if available. The error bars, on the group average, represent the standard deviation.

Individual and group results for speech intelligibility in quiet are shown in Fig 4.

On a group level, based on mean results, there was a tendency of higher intelligibility with SCORE for the conditions CI+HA at 65 dBA, CI only at 50 dBA, HA only at 50 dBA, and HA only at 65 dBA, with differences of +6%, +4%, +10%, and +2%, respectively. However, there was also a tendency of degradation in performance for CI+HA at 50 dBA (-3%) and for CI only at 65 dBA (-4%).

The ANOVA model showed a significant main effect of listening condition (*p* < 0.001), a significant effect of stimulation level (*p* = 0.027), and no effect of processing configuration (*p* = 0.526). Furthermore, there was no significant interaction between factors.

#### Speech in noise

**Fig 5:**
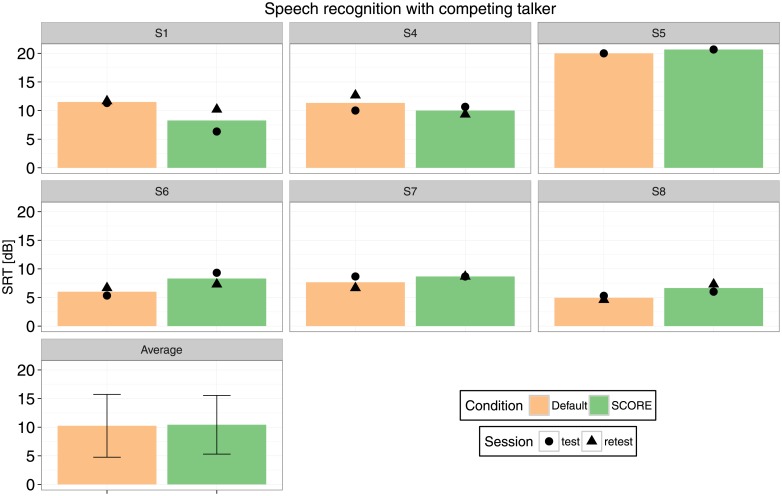
Speech intelligibility in noise. Dots and triangles indicate test and retest results, if available. The error bars on the group average represent the standard deviation.

Individual and group results for speech intelligibility in noise are shown in Fig 5.

There was no significant difference in bimodal speech perception between the conditions with and without SCORE (*p* = 0.684), with a 95% confidence interval of the difference from −2.16 to +2.33 dB. The confidence interval is approaching the standard deviation of the LIST material for normal hearing listeners [30].

#### Localisation

**Fig 6:**
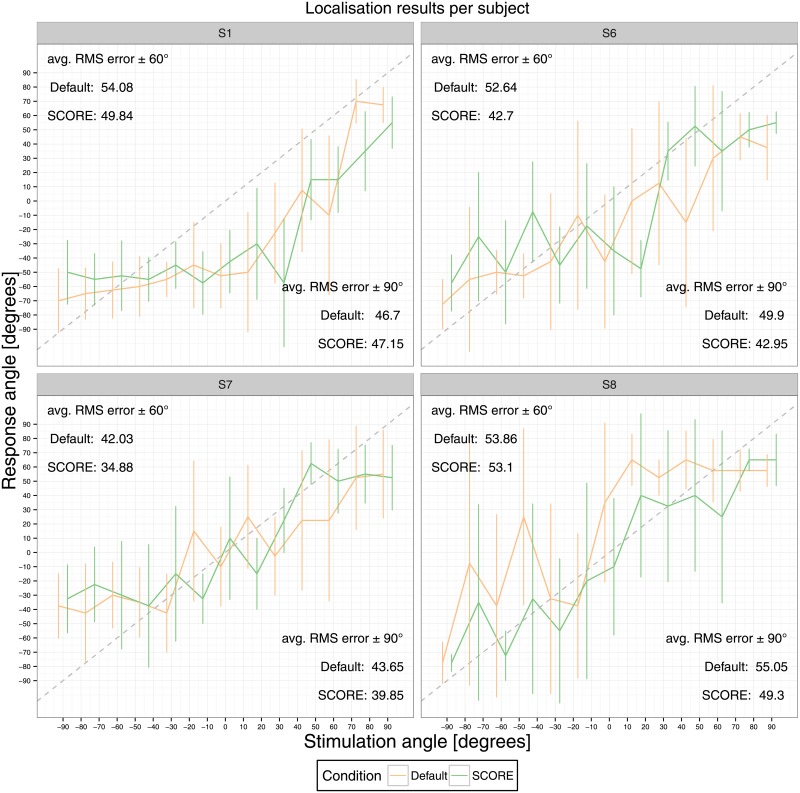
Localisation results. The perceived sound location is depicted per stimulus angle, averaged over presentations. Error bars indicate the standard deviation. The differences between stimulation and perceived angle were used to compute root mean square (RMS) errors, both from angles ±60 and ±90 degrees.

The individual results for the localisation experiment are shown in Fig 6.

**Fig 7:**
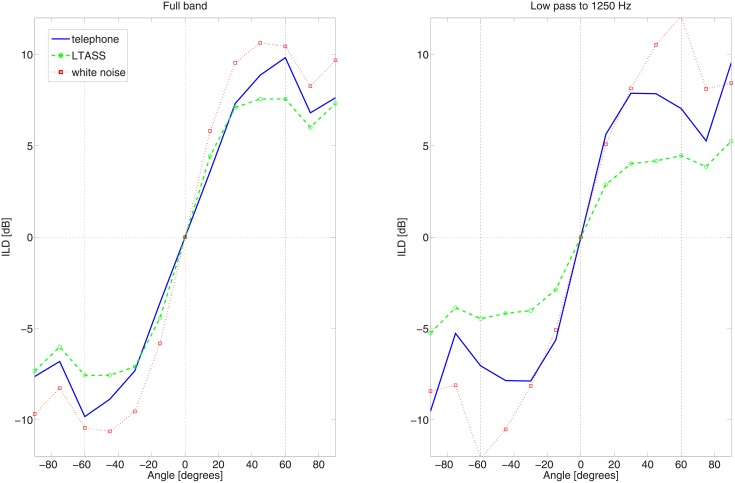
Physically available interaural level differences. The differences are based on the signals at the frontal microphone of behind the ear devices.

Localisation performance improved with SCORE for three out of four subjects by 1 to 7 degrees. In general, localisation performance degraded for angles larger than ±60 degrees. We investigated this effect by computing the physically available ILD, for different stimuli. In the model we used the data for head related transfer function for behind the ear microphones [38]. The ILD as a function of angle is shown in Fig 7.

Fig 7 demonstrates that for the used telephone signal ILDs become ambiguous from 60 degrees. In the case of low frequency residual hearing, only the low frequency part of the stimulus is available and ambiguity will start from smaller angles. In Fig 6 we also show the mean RMS localisation errors for the range of −60 to +60 degrees. For that situation, an advantage for SCORE of up to 10 degrees was observed, with an average of 5 degrees.

In summary, SCORE did not affect speech in quiet and in noise but did improve individual localisation performance.

## Experiment 2: Changing loudness growth from normal

The goal of experiment 2 was to further test SCORE with a compressive HA and with different loudness targets.

### Methods

For conciseness, we will describe only the differences to experiment 1.

#### Subjects

Unaided thresholds were measured again since 10 to 11 months passed between the two experiments.

#### Equipment

We used a hearing aid model with compression from GN Resound (GN Group Nord A/S) [39], which we implemented as a Simulink model. The hearing aid was fitted according to the NAL-NL2 rule [15].

In addition to the overall gain applied by SCORE, we implemented a low-pass filter to reduce the gain for frequencies where hearing thresholds were worse than 100 dBHL.

For the CI model, we activated the automatic gain control (AGC) in order to be closer to clinical practice, compared to experiment 1. Also, we used different time constants for SCORE. To avoid audible artefacts, SCORE gradually applies changes with a certain attack and release time. In experiment 1, SCORE used attack and release times of 5 and 50 ms, respectively. To avoid interactions with the AGC of the two devices, for experiment 2, we used attack and release times of 200 and 50 ms, respectively. For the localisation experiment, attack and release times of, respectively, 100 and 20 ms were used, because the stimulus was only 360 ms long.

**Fig 8:**
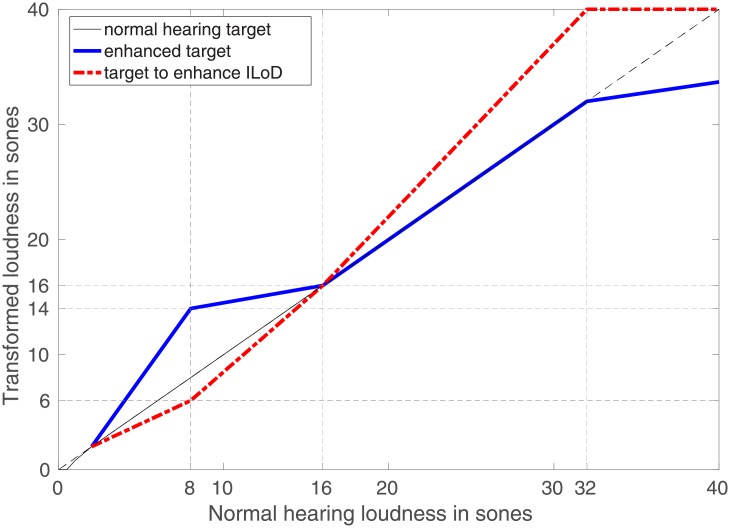
Loudness targets manipulation.

In order to study the influence of target loudness on speech intelligibility and localisation we manipulated the loudness targets at the two sides as shown in Fig 8. We assumed that 16 sones corresponds to a comfortable loudness. Simulations showed that consonants provide loudness around 8 sones and vowels around 16 sones. In experiment 1 the loudness target follows the diagonal up to 32 sones, after which it was limited to avoid loudness discomfort. We created two new targets. The goal of the enhanced target was to make softer sounds louder in order to improve the audibility of the consonants. We matched 8 sones from the normal hearing loudness model to a target of 14 sones. We also introduced a target to enhance interaural loudness differences (ILoD) that should improve localisation. Therefore, we matched 8 and 32 sones from the normal hearing loudness model to targets of 6 and 40 sones, respectively.

In Fig 8, all targets include the 16-sones point of the diagonal because we assumed that 16 sones corresponds to comfortable loudness.

#### Stimuli

Speech perception in quiet was measured at 65 dBA. For the localisation experiment we decreased the level of the stimulus from 65 to 60 dB A in order to avoid saturation due to the compression in the CI and HA models. As in experiment 1, we applied a fixed roving of ±5 dB. Since the level of the stimulus was 5 dB lower, we increased the duration of the stimulus from 250 ms to 360 ms, which is sufficient for loudness integration [40] but still short enough to prevent effective head movements.

#### Procedures

Speech perception in quiet was measured in three binaural configurations (default fitting, SCORE with normal loudness growth, SCORE with enhanced loudness growth) and two CI-only configurations (default fitting and SCORE with enhanced loudness growth). First, we tested the binaural conditions and then the CI-only conditions. In order to measure the intra-subject variability, we tested each binaural configuration three times in a row and each CI configuration two times in a row. To let the subjects get used to the procedure, we used one NVA list for training in one randomly selected binaural condition.

Speech perception in noise was tested in the above-mentioned binaural configurations. Each configuration was tested until results were decreasing, in order to account for learning effects. The order of the tested conditions was random across subjects.

In the localisation experiment we included two conditions (bimodal default fitting and bimodal with SCORE enhanced ILoD). These conditions were also tested in a random order. We tested each condition until the results were decreasing.

### Results

#### Speech in quiet

**Fig 9:**
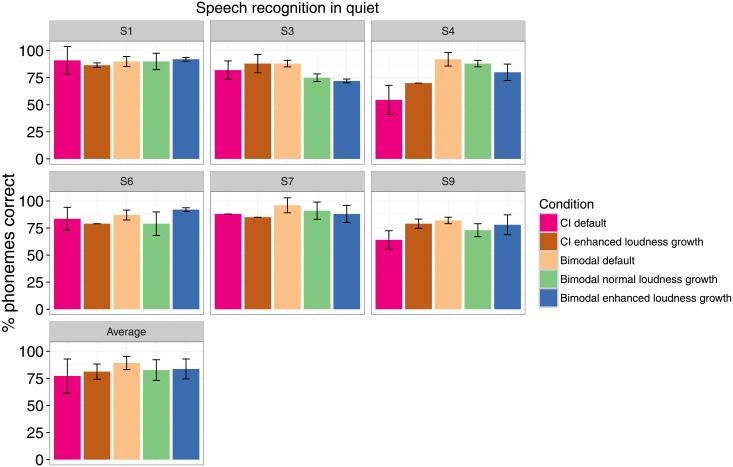
Speech intelligibility in quiet. Error bars indicate the standard deviation across test repetitions (individual results) or across subjects (average results).

Individual and group results for speech intelligibility in quiet are shown in Fig 9.

The ANOVA model on the CI-only results, transformed to RAU, showed no effect of configuration (default or enhanced SCORE, (*p* = 0.73)), no effect of test phase (*p* = 0.64), and no interaction between factors (*p* = 0.92).

The ANOVA model on the bimodal results, transformed to RAU, showed no effect of configuration (default, normal SCORE or enhanced SCORE (*p* = 0.09)), no effect of test phase (*p* = 0.92), and no interaction between factors (*p* = 0.78).

The intra-subject standard deviation, averaged across subjects and conditions, was 5.5% (with a maximum of 13.4%).

#### Speech in noise

**Fig 10:**
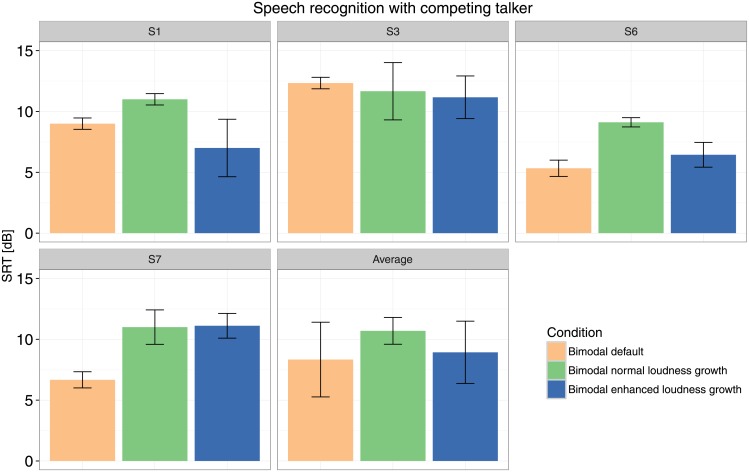
Speech intelligibility in noise. Error bars indicate the standard deviation across test repetitions (individual results) or across subjects (average results).

Individual and group results for speech intelligibility in noise are shown in Fig 10.

The ANOVA model on the SRT results showed no effect of configuration (default, normal SCORE or enhanced SCORE, *p* = 0.07).

#### Localisation

**Fig 11:**
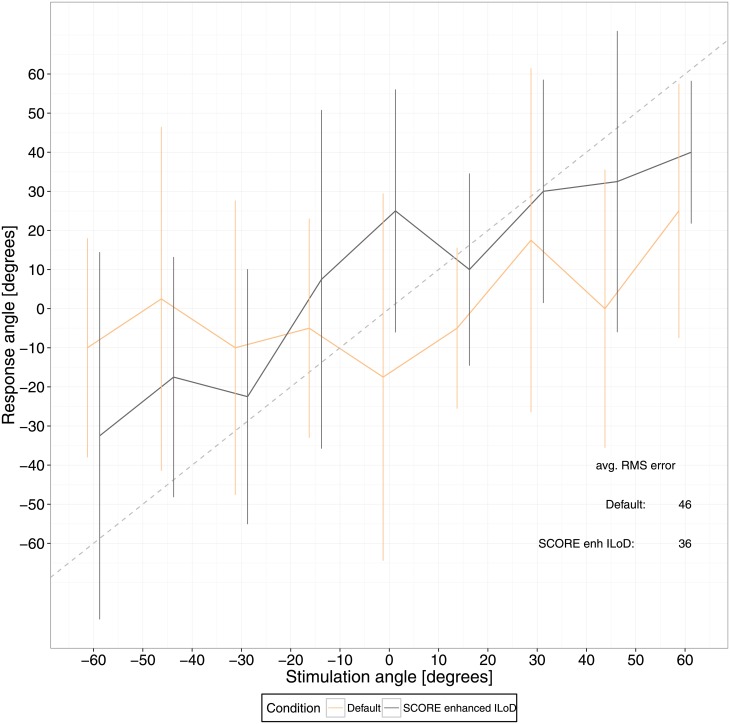
Localisation results. The perceived sound location is depicted per stimulus angle, averaged over presentations. Error bars indicate the standard deviation.

Localisation outcomes are shown in Fig 11.

Based on RMS localisation error, enhancing ILoD improved the localisation performance of this subject by 10 degrees.

In summary, when using manipulated loudness targets no effects where found on speech intelligibility in quiet and in noise, but localisation performance was promising, improving by 10 degrees, with enhanced ILoD.

## General discussion

We implemented a loudness normalisation algorithm on a real-time system and validated it in bimodal users. We simplified the acoustical loudness model. In the majority of cases, for speech or speech-like stimuli, the simplifications led to loudness differences smaller than 3% which should not have a perceptual effect, since these differences are smaller than the just noticeable loudness difference [31].

For speech in quiet the differences in results never reached significance, presumably, due to intra- and inter-subject variability. Also, there was no significant difference for speech in noise. Improvements in sound localisation of up to 10 degrees were observed with SCORE when considering presentation angles from +60 to −60 degrees.

### Simplification of the acoustical loudness model

Using a bins summation, for most speech stimuli, we achieved loudness differences similar to some more complex algorithms. For example Ward [13] found average short term loudness differences from 0.7 to 2.1%, while we achieved instantaneous loudness difference in the range of 0.09 to 2.65% with one outlier at 4.8%. However, it is difficult to compare with other studies [12, 41] since they used different metrics.

### Speech intelligibility in quiet and in noise

Except for the bimodal condition at 50 dB A, the results from this study are consistent with those of Francart and McDermott [11]. In this study they found on average 5 percentage points better speech intelligibility with SCORE in quiet and no difference in noise. Small differences in the applied methods can explain the different results. First, we used free field presentation, and we did not control the small spontaneous head movements, while Francart and McDermott used preprocessed stimuli. Second, the subjects tested by Francart and McDermott had better low frequency residual hearing than the subjects of this study. Also, one of the subjects in the present study had strong tinnitus. We had only one participant (S6-from the first experiment) with pure tone average (0,125-0.5 kHz) better than 60 dB, who demonstrated the largest benefit for speech in quiet with the acoustical part of SCORE. Third, the test material of Francart and McDermott contained 50 CNC words per list, while NVA lists had only 12 CNC words per list (of which the first word is not scored). This might explain the larger test-retest variability in this study. Finally, Francart and McDermott used a full acoustical loudness model while we used a simplified one.

### Localisation

The results are consistent with those of Francart and McDermott [11], where they found 2 to 9 degrees improvement in the localisation with SCORE. In this study, the improvement in sound localisation observed with SCORE was larger for subjects who had better high frequency residual hearing. Simulations also showed that localisation results will depend on the used stimuli and the subject’s residual hearing. By normalising the loudness growth functions SCORE restored the ILoD across ears. In a previous study, where the physically available ILD were enhanced, resulted in localisation improvement of 4 to 10 degrees, in terms of absolute error [35]. Therefore, the ILD and the resultant ILoD are important localisation cues.

### Changing loudness growth

It seems that matching the loudness growth curves from the two devices is more important for localisation performance than for speech intelligibility. From a previous study [18] we know that speech intelligibility in quiet as a function of interaural level balance has a plateau. In other words, it is sufficient that the devices are roughly balanced at a comfortable level to obtain optimal speech intelligibility. Moreover, bimodal listeners have larger just noticeable differences in loudness than normal hearing [42], which, in some extreme cases can be more than 6 dB. This might explain why we did not find a significant effect of SCORE with different loudness growth functions on speech intelligibility. Other factors might be the lack of fusion between the electrical and acoustical stimulation or the lack of adaptation with the changed loudness growth. The default condition and the associated loudness growth are expected to be closer to the subject’s everyday fitting. Therefore, home trial periods are needed to fully investigate an algorithm that changes bimodal loudness growth.

Matched loudness growth seems to be more important for sound localisation, possibly because for localisation the only available cue are ILoDs, while for speech intelligibility mostly other cues are used.

## Conclusion

The present studies led to three main conclusions. First, we successfully implemented an acoustical loudness model with a fixed delay in a real-time system with differences to the original model that should not have a perceptual effect. Second, the preliminary results with SCORE suggest improved sound localisation without influencing speech intelligibility in quiet and in noise. A balanced loudness percept appeared to be a more important cue for localisation than for speech intelligibility. Further investigation with a larger sample size is required.

While it was hard to show a benefit of SCORE in laboratory tests with our current cohort of listeners with poor residual hearing, there may be additional benefits that can only be demonstrated in take-home tests. It is possible that similar loudness growth in the two ears leads to improved wearing comfort and sound quality. Additionally, while we could only demonstrate small improvements in localisation performance, larger improvements could be expected on the one hand for listeners with more residual hearing at high frequencies, where ILDs are large, and on the other hand in combination with other algorithms, such as ILD enhancement [35].
